# “Atypical Glandular Cells” on Cervical Cytology: Correlation Between Glandular Cell Component Volume and Histological Follow‐Up

**DOI:** 10.1002/dc.70033

**Published:** 2025-10-20

**Authors:** Havva Gokce Terzioglu, Alessa Aragao, Julieta E. Barroeta

**Affiliations:** ^1^ Department of Pathology Loyola University Medical Center Maywood Illinois USA; ^2^ Department of Pathology The University of Chicago Chicago Illinois USA

**Keywords:** atypical glandular cells, cut‐off, cyto‐histo correlation, number, Pap smear, quantity

## Abstract

**Background:**

Atypical glandular cells (AGC) in cervical cytology, as defined by the Bethesda System, indicate nuclear atypia beyond reactive changes but without definitive features of malignancy. Although clinically significant because it prompts follow‐up procedures, no quantitative threshold exists for AGC diagnosis. This study evaluated whether the volume of glandular cell clusters (GCC), regardless of atypia, influences AGC interpretation and may contribute to unnecessary sampling.

**Methods:**

Following IRB approval, all cervical cytology cases diagnosed as AGC between January 2014 and June 2024 were retrieved, along with 100 random negative for intraepithelial lesion or malignancy (NILM) cases, and were manually re‐screened with quantification of glandular cell clusters (GCC) defined as a group of ≥ 6 cohesive glandular cells irrespective of origin (endocervical versus endometrial) and the results were correlated with follow‐up findings including endocervical and endometrial sampling.

**Results:**

Of 301 AGC cases, 186 cases had slides available for review and follow‐up data; two were excluded due to unsatisfactory quality. Eight cases were reclassified as unsatisfactory because of insufficient squamous cells and absence of atypia, most of which exhibited high GCC (mean 59). Notably, 140 cases (76.6%) showed no significant glandular pathology on follow‐up, and in 111 cases (60.6%) the follow‐up was negative. Overall, increased GCC correlated significantly with AGC interpretation compared to NILM cases (*p* = 0.01), even when histologic follow‐up was negative.

**Conclusion:**

Higher GCC volumes may influence AGC diagnoses, even in cases lacking true cytologic atypia, potentially leading to unnecessary interventions. Greater awareness of this tendency and adherence to established cytologic criteria may improve diagnostic precision within the AGC category.

## 1 Introduction

The interpretation of atypical glandular cells (AGC) in cervical cytology represents a diagnostic challenge due to the relatively low prevalence of glandular lesions and their broad differential diagnoses, with incidence rates ranging from 0.08% to 2.1% [1, 2, 3, 4, 5, 6, 7, 8, 9, 10, 11, 12, 13, 14, 15].

According to The Bethesda System for Reporting Cervical Cytology, AGC refers to endocervical or endometrial cells exhibiting nuclear atypia that exceeds what is expected from reactive or reparative changes, but without definitive features of malignancy [16]. Despite its rarity, the clinical significance of an AGC diagnosis is considerable, as it typically prompts comprehensive follow‐up work‐up, including colposcopy with endocervical and endometrial sampling, regardless of high‐risk human papillomavirus (HPV) status [17].

There is no established quantitative threshold that defines AGC, and the cytomorphologic interpretation remains largely subjective. As such, various cytologic features, including nuclear enlargement, irregularity, hyperchromasia, and architectural crowding, are considered, but there is no consensus on the weight of glandular cell quantity in the diagnostic process. In practice, overrepresentation of glandular elements in otherwise negative or equivocal smears may lead to a heightened index of suspicion and possible overdiagnosis.

The aim of this study was to investigate whether the volume of glandular cell clusters (GCC), independent of cytologic atypia, influences the interpretation of AGC in cervical cytology. By correlating GCC quantification with histologic follow‐up outcomes, we sought to assess whether glandular cell volume plays a role in triggering an AGC diagnosis and whether this has clinical relevance in guiding patient management.

## 2 Materials and Methods

With approval from the institutional review board, a 10‐year retrospective search of the electronic database was conducted, reviewing cervical Papanicolaou (Pap) tests diagnosed as AGC, not otherwise specified (NOS) between January 2014 and June 2024. Cases were included if cytology slides were available for re‐evaluation and corresponding histologic follow‐up data could be obtained. In addition, 100 cases previously diagnosed as negative for intraepithelial lesion or malignancy (NILM), all of which had undergone secondary screening by cytopathologists and had adequate sampling of the transformation zone, were randomly selected and included as a control group. All slides were independently re‐reviewed by a cytopathologist with over 18 years of experience, who was blinded to patients' clinical information, including age, histologic follow‐up results, and human papillomavirus (HPV) status.

GCC was defined as groups of more than six cohesive glandular cells, irrespective of their endocervical or endometrial origin and regardless of the presence or absence of cytologic atypia. The number of GCCs was quantified in each case and subsequently correlated with the available histologic follow‐up findings to assess potential diagnostic significance.

Histologic follow‐up data included a range of procedures such as endocervical curettage, cervical, vaginal, and endometrial biopsies, excisional biopsies, curettings, and hysterectomies. In cases where multiple follow‐up procedures were performed, the most severe histologic diagnosis was documented for analysis. Follow‐up results were categorized into three groups: (1) significant findings of glandular origin, including endocervical adenocarcinoma in situ, endocervical adenocarcinoma, vaginal adenocarcinoma, endometrial adenocarcinoma, benign and atypical endometrial hyperplasia, and carcinosarcoma; (2) significant findings of squamous origin, such as cervical intraepithelial neoplasia (CIN) 1–3 and squamous cell carcinoma; and (3) no significant pathology, encompassing reactive changes, microglandular hyperplasia, and endocervical or endometrial polyps.

All Pap tests collected during the study period were liquid‐based cytology (ThinPrep^R^, Hologic, Marlborough, MA) and the staining of the slides was performed with Papanicolaou stain using the, Tissue‐Tek DRS 2000 (Sakura Finetek^R^, Torrance, CA.)

All statistical analyses were performed using SPSS software (IBM Corp., Armonk, NY). The Mann–Whitney U test and Kruskal–Wallis test were utilized for non‐parametric comparisons. A *p*‐value of less than 0.05 was considered statistically significant.

## 3 Results

**FIGURE 1:**
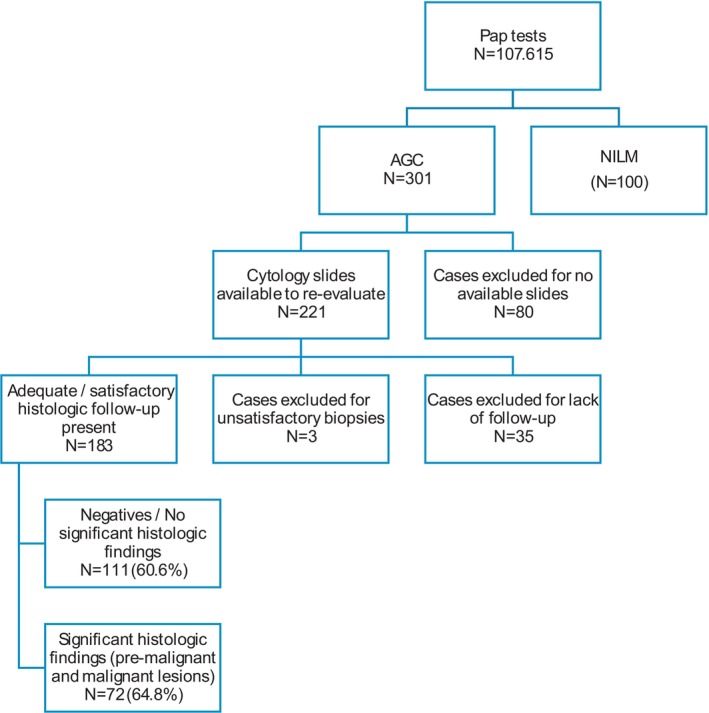
Case selection; atypical glandular cells (AGC) cases with their follow‐up information and randomly selected negative for intraepithelial lesion or malignancy (NILM) cases as a control group.

Over an approximately 10‐year period from January 2014 to June 2024, a total of 107,615 Pap tests were evaluated at our institution—a large academic medical center in an urban setting—among which 301 cases (0.28%) were interpreted as AGC. All these cases had been interpreted as AGC during screening by the cytotechnologists, followed by secondary screening by experienced cytopathologists. These diagnoses were made by 11 board‐certified cytopathologists, with varying levels of experience: Seven had over 10 years of independent practice, and four had fewer than 5 years. Experience was calculated based on the number of years each pathologist had been practicing independently at the time of their appointment at our institution. Of the 301 AGC cases, 253 patients (84%) underwent histologic follow‐up. Cytology slides were retrievable for 221 cases, and 186 of those had corresponding histologic follow‐up. Three follow‐up specimens were excluded from analysis due to unsatisfactory diagnostic quality, attributed to abundant mucin and absence of tissue fragments. Ultimately, 183 cases with available cytology slides and satisfactory histologic follow‐up were included in this study. An additional 100 cases diagnosed as negative for intraepithelial lesion or malignancy (NILM), all with secondary cytopathologist review, were included as controls (Figure 1).

**FIGURE 2:**
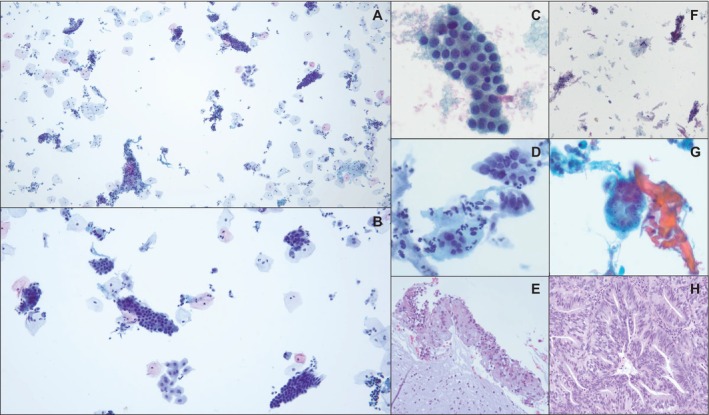
Representative images of glandular cell clusters (GCC) in Pap tests initially diagnosed as atypical glandular cells (AGC), with corresponding histologic follow‐up in selected cases. (A, B) Low‐power views showing an abundance of glandular cell clusters in ThinPrep slides (40× and 100×, Papanicolaou stain), (C) GCC, defined as groups of > 6 cohesive glandular cells, regardless of their origin or atypia (400×, Papanicolaou stain), (D) Endocervical cell cluster with abundant admixed neutrophils, interpreted as AGC, favoring endocervical origin. GCC volume is 242 (400×, Papanicolaou stain), (E) Corresponding biopsy from the same patient as in (D), showing reactive endocervical epithelium with chronic inflammation, consistent with chronic cervicitis (200×, H&E stain), (F) Initially diagnosed as AGC, reclassified as unsatisfactory for evaluation due to insufficient squamous cell component and lack of atypia. GCC volume is 114. Follow‐up revealed no significant finding (40×, Papanicolaou stain), (G) Atypical endometrial cells with enlarged nuclei, prominent nucleoli, and central lumen‐like formation, interpreted as AGC, not otherwise specified (NOS). GCC volume is 266 (400×, Papanicolaou stain), (H) Corresponding endometrial biopsy showing endometrial adenocarcinoma (200×, H&E stain). [Color figure can be viewed at wileyonlinelibrary.com]

Quantification of GCC was performed for all included cases. GCC was defined as clusters of more than six cohesive glandular cells, regardless of cell origin (endocervical/endometrial) or atypia. A cut‐off of six cells was selected because it was identified as a possible number of cells that could trigger a subconscious bias toward the interpretation of AGC. Since there were no other similar prior studies, there was no previously set cut‐off used for comparison. The mean, median, and range of GCC counts are summarized in Table 1. Among the AGC group, eight cases were reclassified as unsatisfactory due to insufficient squamous cellularity and absence of cytologic atypia; these cases showed a relatively high mean GCC count of 59 (Figure 2).

**TABLE 1:** Glandular cell clusters (GCC) metrics of NILM cases and AGC cases with histological follow‐up.

	GCC mean	GCC median	GCC range
AGC cases, overall (*n* = 183)	42.97	20	1–406
AGC with significant findings of glandular origin (*n* = 43)	58.78	16	1–406
AGC with significant findings of squamous origin (*n* = 29)	64.20	42	3–384
AGC with no significant findings (*n* = 111)	31.65	19	1–195
NILM cases with no follow‐up (*n* = 100)	22.6	12	0–192

Abbreviations: AGC, atypical glandular cells; NILM, negative for intraepithelial lesion or malignancy.

Age‐stratified analysis of AGC cases showed the following mean GCC values: for patients under 45 years of age, the mean GCC was 54.75 in cases with significant glandular findings (*N* = 4), 43.0 in cases with squamous findings (*N* = 14), and 27.26 in cases with no significant findings (*N* = 38). For patients aged 45 years and older, the mean GCC was 58.25 in cases with significant glandular findings (*N* = 39), 81.6 in squamous findings (*N* = 16), and 33.87 in cases with no significant findings (*N* = 73). Kruskal–Wallis testing showed no statistically significant difference in GCC values when stratified by age group (*p* = 0.085). All cases were originally diagnosed as AGC; however, for the purposes of this study, they were re‐evaluated by the corresponding author, who has additional expertise in gynecologic pathology. During this blinded review, cases were stratified based on the presence or absence of cytologic atypia. Atypia‐stratified analysis showed that cases with cytologic atypia (*N* = 72) had a mean GCC of 45.87 (median 23; range 1–406), while those without atypia (*N* = 112) had a mean GCC of 41.12 (median 17.5; range 1–384). Full summary statistics for both age and atypia groups are provided in Table 2.

**TABLE 2:** Glandular cell cluster (GCC) metrics stratified by age group, follow‐up histologic findings, and presence of cytologic atypia in AGC cases.

	GCC mean	GCC median	GCC range
Age			
< 45 (*N* = 56)			
Significant findings of glandular origin (*N* = 4)	54.75	16	4–183
Significant findings of squamous origin (*N* = 14)	43.0	43	8–108
No significant findings (*N* = 38)	27.26	15	1–158
≥ 45 (*N* = 128)			
Significant findings of glandular origin (*N* = 39)	58.25	16	1–406
Significant findings of squamous origin (*N* = 16)	81.6	49	3–384
No significant findings (*N* = 73)	33.87	20	1–195
Atypia			
Present (*N* = 72)	45.87	23	1–406
Absent (*N* = 112)	41.12	17.5	1–384

Follow‐up histologic findings were categorized as significant glandular pathology (*n* = 42), significant squamous pathology (*n* = 29), or no significant pathology (*n* = 113). There was no statistically significant association between GCC volume and the severity or presence of histologic abnormalities within AGC cases (*p* = 0.222). However, a statistically significant difference in GCC volume was observed between AGC and NILM cases overall (*p* = 0.01), and even when comparing AGC cases with negative follow‐up to NILM controls (*p* = 0.017), suggesting a possible influence of glandular cell volume on AGC interpretation.

## 4 Discussion

The presence of endocervical cells in Pap tests has historically been used as a determinant of sample quality [18], although their presence or absence does not impact adequacy as the squamous component does. The Bethesda System states that “at least ten well‐preserved endocervical or squamous metaplastic cells, singly or in clusters” be identified as a measure of adequate transformation zone sampling [16]. Previous studies have analyzed the impact of the presence or absence of endocervical cells and detection of cervical abnormalities [19]. Some studies have suggested that the presence of endocervical cells correlates with squamous cervical abnormalities, while other studies have found no association [6, 20, 21, 22, 23]. In one study, only the presence of metaplastic cells but not endocervical cells correlated with the presence of cervical intraepithelial neoplasia [24]. While prior research has primarily focused on the presence or absence of the endocervical cell component, our daily experience in cytology sign‐out raised the question of whether the volume of glandular cells, independent of their cytologic atypia, influences the interpretation of AGC.

In the study by Ribeiro et al., the authors analyzed the relation between endocervical cell counts (total number of individual endocervical cells) and the presence of squamous cell abnormality, including LSIL and HSIL, in conventional Pap smears. Their results demonstrated a significant association of higher numbers of endocervical cells with the detection of squamous cell abnormalities [25]. The authors developed a scoring system to quantify endocervical cells, with smears categorized into five groups based on increasing cell counts. Their findings demonstrated a significant association between higher endocervical cell scores and the presence of ≥ 10 atypical squamous cells, with smears containing more than 50 endocervical cells showing an odds ratio of 2.87 for the presence of squamous atypia compared to those with ≤ 5 cells [25].

These results suggest that not only the presence but the quantity of endocervical cells may enhance the visibility or detection rate of squamous abnormalities. This likely reflects more complete sampling of the transformation zone, where most squamous lesions arise, rather than diagnostic bias. However, a similar relationship between glandular cell volume and detection of glandular abnormalities has not been established. Unlike squamous lesions, glandular abnormalities are less common and may present with subtler cytologic features.

In the absence of definitive cytologic features required for an AGC diagnosis, such as nuclear enlargement and chromatin abnormalities, cases with a high number of glandular clusters may still be more prone to AGC interpretation due to potential interpretation bias. Our results demonstrated a significant correlation between increased GCC volume and AGC interpretation as compared to NILM cases. There were significant differences in the GCC means between AGC cases with confirmed abnormal findings of glandular origin (*n* = 43, GCC mean: 58.78) or squamous origin (*n* = 29, GCC mean: 64.2) when compared to cases interpreted as NILM (*n* = 111, GCC mean: 22.6). Interestingly, in cases diagnosed as AGC without any significant abnormal findings on histological correlation, the mean GCC was 31.65, which was significantly different from the mean GCC of NILM smears (*p* = 0.017).

Although age and menopausal status are known to influence cervical cytology interpretation, our age‐stratified analysis did not reveal a statistically significant difference in GCC values between patients younger and older than 45 across follow‐up categories. This finding suggests that while glandular cell volume may vary with clinical context, it alone does not correlate strongly with age, reinforcing the need to prioritize established morphologic criteria in AGC interpretation.

An additional finding was the subset of eight cases initially diagnosed as AGC that were deemed unsatisfactory upon re‐evaluation due to insufficient squamous cellularity and absence of cytologic atypia. Interestingly, these cases exhibited a disproportionately high glandular component, with a mean GCC of 59. Despite this finding, follow‐up revealed no significant glandular abnormalities in 76.5% of cases (*n* = 140, either negative or significant squamous findings on histologic follow‐up), and 60.6% had entirely negative histologic findings (*n* = 111). This further underscores the potential for overdiagnosis when high glandular cellularity is present in the absence of diagnostic atypia. It also highlights the importance of adequate squamous representation and adherence to strict cytologic criteria when rendering AGC diagnoses. Without a sufficient squamous component, cytologists and cytopathologists may place undue diagnostic weight on glandular elements, especially in cases with prominent but benign‐appearing glandular cells. These findings may be attributable to a cognitive bias where an abundance of glandular elements raises concern for a glandular lesion, prompting an AGC designation independent of the presence of definitive features to establish such diagnosis. The lack of standardized quantitative criteria in The Bethesda System for AGC interpretation allows for subjectivity in borderline cases. As such, cytologists and cytopathologists may adopt a more cautious diagnostic approach, especially when faced with abundant glandular cells in otherwise equivocal smears. This has implications for patient care, as an AGC diagnosis initiates a cascade of follow‐up procedures, including colposcopy, endocervical curettage, and potentially endometrial sampling, interventions that are often invasive, stressful, and costly.

Our data emphasize the importance of distinguishing between glandular cell quantity and cytologic quality. While a high number of glandular cells may reflect a well‐sampled transformation zone or hormonal influences, their presence alone should not be the primary basis for an AGC diagnosis. Emphasis should remain on identifying established cytologic features of glandular atypia, such as nuclear enlargement, stratification, hyperchromasia, and abnormal architecture.

A potential limitation of this study is its retrospective design and reliance on cases from a single academic institution, which may affect the generalizability of the findings. Additionally, while cytologic review was blinded to follow‐up and HPV status, other subtle interpretive factors, such as cellularity or background findings, may have influenced the original diagnosis. Further multicenter studies incorporating interobserver variability and HPV stratification may provide additional insights. Multi‐institutional studies with larger cohort groups as well as studies analyzing the impact of GCC in other diagnostic categories would be useful in the replication and confirmation of our findings and could further expand on our understanding of this topic.

In summary, our results suggest that increased GCC volume may play a role in the interpretation of AGC, even in cases lacking definitive cytologic atypia. This observation highlights the potential for interpretive variability when glandular cellularity is elevated. Increasing awareness of this diagnostic tendency, along with consistent application of established morphologic criteria, may help minimize unnecessary follow‐up procedures and enhance diagnostic accuracy specifically within the AGC category of cervical cytology.

## Conflicts of Interest

The authors declare no conflicts of interest.

## Data Availability

The data that support the findings of this study are available from the corresponding author upon reasonable request.
